# How the host becomes the target: exploiting an intracellular transport pathway to treat coronaviruses

**DOI:** 10.1172/JCI203655

**Published:** 2026-03-02

**Authors:** Pablo Penaloza-MacMaster

Viruses rely on both viral and host proteins to assemble and release progeny virions. Most antiviral therapies, including viral enzyme inhibitors and monoclonal antibodies, have focused on the virus itself, and host-directed therapies have remained understudied (1–3). In this issue of the *JCI* (4), investigators identified hepatocyte growth factor-regulated tyrosine kinase substrate (HGS), a host intracellular trafficking protein, as a conserved and druggable target critical for coronavirus virion assembly.

Using a CRISPR inhibition screen, Long et al. pinpointed HGS as a central regulator of coronavirus assembly (4). Mechanistic studies revealed that HGS engages with the viral membrane (M) protein, which is a viral structural protein important for coronavirus assembly (5–7). Interestingly, the investigators showed that pharmacologic disruption of the host HGS–viral M protein interface reduces virion formation, potentially serving as a target for antiviral therapy.

A notable strength of the study is its translational potential. Peptides derived from the M protein were shown to act as decoys, reducing viral burden in vivo. In parallel, the authors screened more than 5,000 FDA-approved or commonly used compounds and identified riboflavin tetrabutyrate (RTB) as a small-molecule inhibitor that binds HGS and disrupts its interaction with the coronavirus M protein. RTB exhibited antiviral activity against various coronaviruses, supporting its potential utility as a pan-coronavirus therapeutic (4).

One of the most elegant aspects of this study is the tissue-specific genetic validation of HGS function in vivo. Long et al. selectively inactivated HGS in the liver by administering a liver-tropic adeno-associated virus to silence the gene coding for HGS. Such an approach resulted in efficient reduction of HGS expression in the liver but not in the lung. Following intranasal infection with mouse hepatitis virus, viral titers were markedly reduced in the liver but not in the lung of mice with liver-specific HGS silencing. Histological analyses also revealed significantly attenuated liver pathology in these animals (4). Together, these results provide compelling in vivo evidence that HGS is a critical, tissue-intrinsic host factor supporting coronavirus infection.

This work illustrates the promise of targeting conserved host components as antiviral therapeutics. Moving forward, the study’s demonstration that HGS supports virion assembly across divergent coronaviruses raises important opportunities for pandemic preparedness. Host-directed drugs may complement virus-directed drugs, such as polymerase and protease inhibitors. This study provides both a mechanistic foundation and a therapeutic blueprint for host-directed antivirals that are urgently needed, as coronaviruses continue to infect and evolve in the human population.

## Version 1 03/02/2026

Electronic publication
